# Self-rated health and its determinants among adults in Syria: a model from the Middle East

**DOI:** 10.1186/1471-2458-7-177

**Published:** 2007-07-25

**Authors:** Taghrid Asfar, Balsam Ahmad, Samer Rastam, Tanja P Mulloli, Kenneth D Ward, Wasim Maziak

**Affiliations:** 1 Syrian Centre for Tobacco Studies, Aleppo, Syria; 2 Institute of Health and Society, Newcastle University, Newcastle, UK; 3 Department of Health and Sport Sciences, and Center for Community Health, The University of Memphis, Memphis, TN, USA; 4 Institute of Epidemiology and Social Medicine, University Clinic Muenster, Muenster, Germany

## Abstract

**Background:**

Self-rated health (SRH) has been widely used to research health inequalities in developed western societies, but few such studies are available in developing countries. Similar to many Arab societies, little research has been conducted in Syria on the health status of its citizens, particularly in regards to SRH. This Study aims to investigate and compare determinants of SRH in adult men and women in Aleppo, Syria.

**Methods:**

A cross-sectional survey of adults 18 to 65 years old residing in Aleppo (2,500,000 inhabitants), Syria was carried out in 2004, involving 2038 household representatives (45.2% men, age range 18–65 years, response rate 86%). SRH was categorized as excellent, normal, and poor. Odds ratios for poor and normal SRH, compared to excellent, were calculated separately for men and women using logistic regression.

**Results:**

Women were more likely than men to describe their health as poor. Men and women were more likely to report poor SRH if they were older, reported two or more chronic health problems, or had high self perceived functional disability. Important gender-specific determinants of poor SRH included being married, low socioeconomic status, and not having social support for women, and smoking, low physical activity for men.

**Conclusion:**

Women were more likely than men to describe their health as poor. The link with age and pre-existing chronic conditions seems universal and likely reflects natural aging process. Determinants of SRH differed between men and women, possibly highlighting underlying cultural norms and gender roles in the society. Understanding the local context of SRH and its determinants within the prevailing culture will be important to tailor intervention programs aimed at improving health of the Syrian and similar Arab societies.

## Background

Self-rated health (SRH) has been widely used to research health inequalities in developed western societies, but few such studies are available in developing countries. Similar to many Arab societies, little research has been conducted in Syria on the health status of its citizens, particularly in regards to SRH.

The Syrian society is experiencing rapid lifestyle changes resulting in a double burden of disease: Chronic diseases such as diabetes and coronary heart disease are on the increase while infectious diseases still constitute a major cause of mortality and morbidity [1]. Mortality and morbidity indicators in Syria are similar to those seen in other low to middle income countries. Similar to many developed and developing countries, life expectancy at birth in Syria is higher for females than males (74 compared to 69 years respectively) [2]. Recent evidence suggests that smoking and obesity are already highly prevalent in the Syrian society. The Syrian Centre for Tobacco Studies (SCTS) documented in 2006 that about half of Syrian men and one fifth of women currently smoke cigarettes [3]. A recent study from the city of Aleppo shows that obesity is highly prevalent affecting almost half of women studied [4].

SRH has been shown to be a valid and reliable indicator for overall morbidity [5] and a good predictor of mortality [6-8]. Determinants of SRH in men and women fall into a range of domains: socio-demographic, e.g. age and employment [9,10]; diagnosed chronic health conditions [11]; psychological factors, e.g. distress [12]; social support, e.g. social network, social support [13]; and health behaviours and health risk factors [14]. Evidence from developing and developed countries suggests that women tend to report more ill health than men. These disparities appear to be most pronounced in developing countries (i.e. Pakistan) [15] and countries in transition (i.e. Ukraine) [16].

Here we present the first evidence of the spread of poor SRH as well as gender differences in SRH and its determinants in an Arab society.

## Methods

### Design and procedures

The Aleppo Household Survey was conducted between May-August 2004 and targeted adults aged 18 to 65 years residing in the greater city of Aleppo (around 2,500,000 inhabitants). The survey was administered by an interviewer using a personal computer-interface, and included eight sections covering socio-demographics, general health and disability, chronic disease, respiratory health, household members' health, environmental health, smoking, and environmental tobacco exposure. Anthropometric measures (weight, height) also were obtained at the time of the interview. The questionnaire domains used in this study were mostly based on WHO's World Health Survey [1].

The sample consisted of 2,038 household representatives, 45.2% men (mean age + SD 36.4+12.1 years) and 54.8% women (mean age +SD 34.3+11.9 years). The response rate was 86%. The age distribution of the sample was similar to that of adults (18–65) in the general population according to the 2004 census [17]. However, females were slightly over represented in the sample due to their increased availability at home. A detailed description of the sampling, design and procedures of the survey has been reported elsewhere [1]. Briefly, stratified cluster sampling was used to stratify residential neighbourhoods into formal and informal (areas built without approval from municipal authorities) based on the official description of the municipal registry. For both strata neighbourhoods were randomly selected with probability proportional to population size (PPS). Within each chosen neighbourhood, households were selected with equal probability and an adult was interviewed from each household. The protocol and the informed consent documents were approved by the Institutional Review Boards at the University of Memphis and the SCTS.

### Outcome measure

Information on the dependent variable, SRH, was obtained by asking respondents the question: "Generally how do you describe your own health: excellent, good, normal, bad, or very bad". Responses were categorised into excellent (excellent, good), normal, and poor (bad, very bad).

### Explanatory variables

Explanatory variables were examined across four domains: socio-demographic characteristics, health measures, health behaviours, and social support.

#### Socio demographic variables

We categorized age, education, work status, and household density (residents/room). A composite score for socioeconomic status (SES score) was constructed (as illustrated in table 1) [1] and scores were divided into three categories along tertile values with higher scores indicating better socioeconomic status.

**Table 1:** variables used to construct the socioeconomic status, and physical activity scores

	value 0	value 1	value 2
**Socioeconomic status score (0–12)**			
Education	Illiterate	≤ 9 years	> 9 years
Employment	Unemployed, student	Employed (manual, private, government), retired	Employer, private business
Items ownership (phone, mobile phone, PC, AC, private car, TV, satellite dish)	≤ 2	3–4	> 4 or private car
Household members with paid job	0	1	> 1
Household self reported monthly income (from all sources)	< 10,000 SL	10,000–20,000	> 20,000
Density index (household/rooms)	≥ 2.3	1.5–2.3	> 1.5
**Physical activity score (0–4)**			
Regular practice of sports	No	Yes (<3 times/week)	Yes (≥3 times/week)
Frequency of >10 minutes walk/past month	None or rarely	1–2 days/week	3 or more days/week

#### Health measures

Information on chronic health problems was collected by asking participants about past year suffering from the following physician-diagnosed conditions: diabetes, hypertension, respiratory disease, heart disease (angina, infarction, and failure), stroke, kidney disease, liver disease, depression, elevated cholesterol, rheumatism, parasites, peptic ulcer, and cancer. Responses were combined into three categories: none, one, and two or more. Information about functional disability and affection was obtained by asking participants about how much difficulty they had in the past year with mobility, self care, aches and pain, back pain, concentrating, personal relationship, vision, sleeping, feeling tired, being depressed, anxiety, and having problem of teeth and gum. The answers were recorded on a four-point scale: 0 = none, mild, moderate, to 3: severe. Summary scores ranged from 0 to 36 points with higher values indicating more severe functional disability. The functional disability score was stratified into tertiles (0 – 9: little disability, 10 – 16: moderate, and 17 – 36: severe disability). Participants' weight and height were measured for the calculation of the body mass index (BMI) [1] scores which were divided into three categories (<25, 25–30 and >30).

#### Health behaviours

We used two measures of health behaviours namely, smoking and physical activity. Information on smoking was obtained by asking participants whether they smoked or not in the past month because smoking was defined on the basis of self-reported past month cigarette or waterpipe at the time of survey [18]. Answers for smoking were dichotomised (yes, no). Information about physical activity was obtained by asking about the frequency of practicing sports (no, ≤ 3 times per week and ≥ 3 times per week) and frequency of more than 10 minute walk per day (none or rarely, 1–2 days/week, 3 or more days per week). We calculated overall scores for physical activity and categorised them into tertiles (low < 1, middle 1–3 and high >3) (as illustrated in table 1).

#### Social support

We collected information on social support using two questions; "*Do you have someone who supports you when needed*?", and "*Do you have someone to share happiness and sorrow with*? Answers to these two questions were dichotomised (yes, no).

### Statistical analysis

Table 2 presents the distribution of a range of socio-demographic characteristics by gender in the study sample. Table 3 presents the distribution of a range of socio-demographic characteristics across the three categories of SRH and stratified by gender in the study sample.

**Table 2:** Main socio-demographics characteristics of the study population according to gender

	**Male n (%)**	**Female n (%)**	**Total n (%)**
**Neighborhood**			
Formal	451 (49.0)	566 (50.7)	1017 (49.9)
Informal	470 (51.0)	551 (49.3)	1021 (50.1)
**Age**			
18–29 years	305 (33.1)	431 (38.6)	736 (36.1)
30–45 years	398 (43.2)	476 (42.6)	874 (42.9)
46–65 years	218 (23.7)	210 (18.8)	428 (21.0)
**Marital status**			
Married	710 (77.1)	834 (74.7)	494 (24.2)
Non-married (single/divorced/widowed)	211 (22.9)	283 (25.3)	1544 (75.8)
**Education**			
Illiterate	128 (13.9)	297 (26.6)	425 (20.9)
Years of education < 9	546 (59.3)	585 (52.4)	1131 (55.5)
Years of education > 9	247 (26.8)	235 (21.0)	482 (23.7)
**Employment categorized**			
Unemployed	95 (10.3)	964 (86.3)	1059 (52.0)
Employed/retired	429 (46.6)	113 (10.1)	542 (26.6)
Employer/private business	397 (43.1)	40 (3.6)	437 (21.4)
**Density index (DI) categorized**			
>2.3	209 (22.7)	256 (22.9)	465 (22.8)
1.5 – 2.3	277 (30.1)	323 (28.9)	600 (29.4)
<1.5	435 (47.2)	538 (48.2)	973 (47.7)
**SES score categorized**			
Low (score 0–3)	180 (19.5)	611 (54.7)	791 (38.8)
Middle (score 4–5)	390 (42.3)	320 (28.6)	710 (34.8)
High (score 6–12)	351 (38.1)	186 (58.2)	537 (26.3)
**Religion**			
Muslim	884 (96.0)	1054 (94.4)	1938 (95.1)
Christian and others	34 (3.7)	61 (5.5)	95 (4.7)
**Ethnicity**			
Arab	730 (79.3)	895 (80.1)	1625 (79.7)
Others	190 (20.6)	219 (19.6)	409 (20.1)

**Table 3:** Main socio-demographics characteristics of the study population (n = 2038) according to Self-Rated Health (SRH) status and stratified by gender

	**Male**			**Female**		
	**Self Rated Health (SRH)**			**Self Rated Health (SRH)**		

	**Excellent n (%)** 590 (52.4)	**Normal n (%)** 274 (37.8)	**Poor n (%)** 57 (30.6)	**Excellent n (%)** 537 (47.6)	**Normal n (%)** 451 (62.2)	**Poor n (%)** 129 (69.4)

**Neighborhood**						
Formal	279 (47.3)	151 (55.1)	21 (36.8)	270 (50.3)	239 (53)	57 (44.2)
Informal	311 (52.7)	123 (44.9)	36 (63.2)	267 (49.7)	212 (47)	72 (55.8)
**Age**						
18–29 years	228 (38.6)	62 (22.6)	15 (26.3)	257 947.9)	147 (32.6)	27 (20.9)
30–45 years	258 (43.7)	120 (43.8)	20 (35.1)	224 (41.7)	200 (44.3)	52 (40.3)
46–65 years	104 (17.6)	92 (33.6)	22 (38.6)	56 (10.4)	104 (23.1)	50 (38.8)
**Marital status**						
Married	436 (73.9)	228 (83.2)	46 (80.7)	363 (67.6)	358 (79.4)	113 (87.6)
Single/Divorced/Widowed	154 (26.1)	46 (16.8)	11 (19.3)	174 (32.4)	93 (20.6)	16 (12.4)
**Education**						
Illiterate	80 (13.6)	32 (11.7)	16 (28.1)	115 (21.4)	120 (26.6)	62 (48.1)
Years of education < 9	345 (58.5)	171 (62.4)	30 (52.6)	287 (53.4)	243 (53.9)	55 (42.6)
Years of education > 9	165 (28)	71 (25.9)	11 (19.31)	135 (25.1)	88 (19.5)	12 (9.3)
**Employment categorized**						
Unemployed	65 (11)	22 (8)	8 (14)	459 (85.5)	387 (85.8)	118 (91.5)
Employed/retired	268 (45.4)	132 (48.2)	29 (50.9)	59 (11)	48 (10.6)	6 (4.7)
Employer/private business	257 (43.6)	120 (43.8)	20 (35.1)	19 (3.5)	16 (3.5)	5 (3.9)
**Density index (DI) categorized**						
>2.3	128 (21.7)	58 (21.2)	23 (40.4)	116 (21.6)	101 (22.4)	39 (30.2)
1.5 – 2.3	175 (29.7)	89 (32.5)	13 (22.8)	155 (28.9)	135 (29.9)	33 (25.6)
<1.5	287 (48.6)	127 (46.4)	21 (36.8)	266 (49.5)	215 (47.7)	57 (44.2)
**SES score categorized**						
Low (score 0–3)	111 (18.8)	51 (18.6)	18 (31.6)	272 (50.7)	237 (52.5)	102 (79.1)
Middle (score 4–5)	249 (42.2) 230 (39)	119 (43.4) 104 (38)	22 (38.6) 17 (29.8)	156 (29.1) 109 (20.3)	145 (32.2) 69 (15.3)	19 (14.7) 8 (6.2)
High (score 6–12)						
**Religion**						
Muslim	564 (95.6)	263 (96)	57 (100)	495 (92.2)	433 (96)	126 (97.7)
Christian and others	26 (4.4)	11 (4.1)	0	42 (7.8)	18 (4)	3 (2.4)
**Ethnicity**						
Arab	477 (80.8)	211 (77)	42 (73.7)	427 (79.5)	365 (80.9)	103 (79.8)
Others	113 (19.2)	63 (23)	15 (26.3)	110 (20.5)	86 (19)	26 (20.2)

The data were analysed by undertaking logistic regression for men and women separately whereby each exploratory variable was entered separately in the univariate logistic regression and then variables showing association below *p *= *0.2 *were entered in a multinomial logistic regression model. Excellent SRH was used a reference category. Results were presented as odds ratios (OR) and 95% confidence intervals (95% CI) were calculated for poor and normal SRH separately. We used SPSS software (version11.0) for the statistical analysis.

## Results

Overall, 55.3% of participants reported excellent SRH compared to 35.6% reporting normal SRH and 9.1% reporting poor SRH. Women were more likely than men to describe their health as poor (69.4% of those reporting poor SRH were women compared to 30.6% men). Participants who were older, married, less educated, and unemployed reported poorer SRH than the rest of the sample.

**Figure 1:**
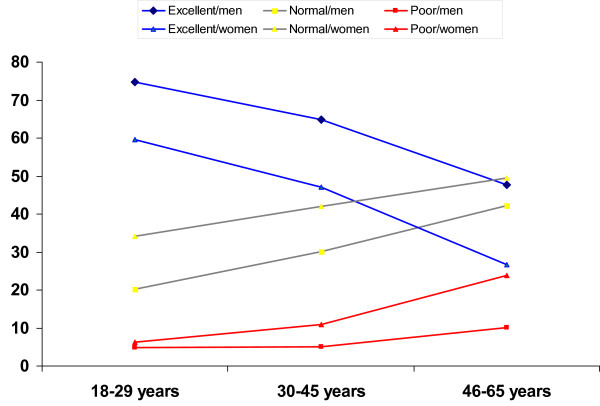
This figure illustrates the prevalence of three categories of SRH (excellent, normal, and poor) according to gender and age of participants.

Univariate logistic regression showed that women in both age groups 30–45 and 46–65 were more likely than men to report poor health (Figure 1). The most important determinants for poor SRH in women were older age, being married, having low education, being unemployed, low socioeconomic status, the existence of chronic conditions, functional disability, high BMI, low physical activity, and lack of someone to share happiness and sorrow (Table 4). For men, the important determinants of poor SRH in the univariate analysis were older age, low education, unemployment, poor socioeconomic status, chronic conditions, functional disability, lack of social support, low physical activity, and smoking (Table 4).

**Table 4:** Determinants of SRH among adult men and women in Aleppo, Syria (n = 2038): odds ratios for normal and poor SRH according to univariate logistic regression

	**Male**		**Female**	
	**Poor/excellent OR (95%CI)**	**Normal/excellent OR (95%CI)**	**Poor/excellent OR (95%CI)**	**Normal/excellent OR (95%CI)**

**Age**				
18–29 years	1	1	1	1
30–45 years	1.2 (0.5 – 2.3)	1.7 (1.2 – 2.4)*	2.2 (1.3 – 3.6)*	1.5 (1.2–2.1)*
46–65 years	3.2 (1.6 – 6.4)*	3.3 (2.2 – 4.8)*	8.4 (4.9 – 14.7)*	3.2 (2.2 – 4.7)*
**Marital status**				
Married	1	1	1	1
Not married	0.7 (0.3 – 1.3)	0.6 (0.4–0.8)*	0.3 (0.1 – 0.5)*	0.5 (0.4–0.7)*
**Education**				
Illiterate	1	1	1	1
Years of education < 9	0.4 (0.2 – 0.8)*	1.2 (0.8 – 1.9)	0.3 (0.2 – 0.5)*	0.8 (0.6 – 1.1)
Years of education > 9	0.3 (0.1 – 0.7)*	1.1 (0.6 – 1.7)	0.2 (0.08 – 0.3)*	0.6 (0.4 – 0.9)*
**Employment categorized**				
Unemployed	1	1	1	1
Employed/retired	0.8 (0.3 – 2)	1.5 (0.8 – 2.5)	0.4 (0.2 – 0.9)*	0.9 (0.6 – 1.4)
Employer/private business	0.6 (0.2 – 1.5)	1.3 (0.8 – 2.3)	1 (0.4 – 2.8)	0.9 (0.5 – 1.9)
**Density index (DI) categorized**		0.9		
>2.34	1	1	1	1
1.5 – 2.34	0.4 (0.2 – 0.8)*	1.1 (0.7 – 1.6)	0.6 (0.4 – 1)	1 (0.7 – 1.4)
<1.5	0.4 (0.2 – 0.7)*	0.10 (0.6 – 1.4)	0.6 (0.4–1.0)	0.9 (0.7 – 1.3)
**SES score categorized**				
Low (score 0–3)	1	1	1	1
Middle (score 4–5)	0.5 (0.2 – 1)	1 (0.7 – 1.5)	0.3 (0.2 – 0.5)*	1.1 (0.8 – 1.4)
High (score 6–12)	0.4 (0.2 – 0.9)*	0.9 (0.6 – 1.5)	0.2 (0.09 – 0.4)*	0.7 (0.5 – 1)
**Chronic conditions**				
None	1	1	1	1
One	1.8 (0.8 – 3.9)	2.7 (1.9 – 3.8)*	5.4 (3 – 9.6)*	2.1 (1.5 – 2.9)*
Two or more	9.7 (5 – 18.5)*	5.2 (3.5 – 78)*	14 (8.1 – 24.4)*	3.6 (2.6 – 5)*
**Self-perceived functional disability and affection**				
0–9 little disability	1	1	1	1
10–16 moderate disability	4.4 (1.7 – 10.9)*	3.3 (2.3 – 4.8)*	7 (2.4 – 20.6)*	3.6 (2.5 – 5.2)*
17–36 sever disability	18.7 (8 – 43.6)*	6.4 (4.3 – 9.5)*	43.3 (15.6 – 120.2)*	7.3 (5 – 10.5)*
**Body mass index**				
<25	1	1	1	1
25–30	0.7 (0.4 – 1.4)	1.1 (0.8 – 1.6)	1.8 (1.03 – 3.4)*	1.1 (0.8 – 1.5)
>30	0.6 (0.3 – 1.3)	1.5 (1.1 – 2.2)*	2.9 (1.7 – 5.2)*	1.6 (1.2 – 2.1)*
**Health behaviour**				
Smoking cigarettes ("no" as reference) Physical activities score (0–4)	2.9 (1.5 – 6)*	1.1 (0.8 – 1.5)	1.2 (0.7 – 1.8)	1.4 (1.1 – 1.9)*
Low < 1	1	1	1	1
Middle (score 1–3)	0.3 (0.2 – 0.6)*	0.5 (0.4 – 0.7)*	1 (0.7 – 1.5)	1 (0.8 – 1.4)
High >3	0.1 (0.03 – 0.3)*	0.4 (0.2 – 0.6)*	0.3 (0.1 – 0.8)*	0.7 (0.4 – 1.1)
**Social support**				
Have someone who supports you when needed ("no" as reference)	0.5 (0.3 – 0.7)*	1 (0.7 – 1.4)	0.5 (0.3 – 0.7)*	0.9 (0.7 – 1.4)
Have someone to share with happiness & sorrow ("no" as reference)	1 (0.4 – 2.3)	0.8 (0.5 – 1.4)	0.4 (0.2 – 0.7)*	0.8 (0.6 – 1.3)

*p <*0.05 *according to univariate logistic regression analysis.

Table 5 shows the results of analysis from the multinomial logistic regression. The results show that older age remained an important predictor or poor SRH in both men and women. Other factors remained distinguishing women and men determinants of SRH were being married, low socioeconomic status, and not having social support for women, and smoking, low physical activity for men.

**Table 5:** Determinants of SRH among adult men and women in Aleppo, Syria (n = 2038): odds ratios for normal and poor SRH according to multinomial logistic regression

	**Male**		**Female**	
	**Poor/excellent**	**Normal/excellent**	**Poor/excellent**	**Normal/excellent**

**Age**				
18–29 years	1	1	1	1
30–45 years	0.8 (0.3 – 1.9)	1.5 (0.9 2.4)	1.5 (0.8 – 2.7)	1.2 (0.8 – 1.7)
46–65 years	2.9 (1.1 – 7.7)*	2.7 (1.6 – 4.6)*	4.1 (1.9 – 8.4)*	2.3 (1.4 – 3.8)*
**Socio- economic score**				
Low (score 0–3)	1	1	1	1
Middle (score 4–5)	0.6 (0.2 – 1.2)	1.05 (0.7 – 1.6)	0.43 (0.2 – 0.8)*	1.3 (0.9 – 1.70
High (score 6–12)	0.5 (0.2 – 1.2)	0.9 (0.6 – 1.5)	0.3 (0.1 – 0.8)*	0.9 (0.7 – 1.4)
**Education**				
Illiterate	1	1	1	1
Years of education < 9	0.5 (0.3 – 1.2)	1.4 (0.8 – 2.3)	0.9 (0.5 – 1.5)	1.2 (0.9 – 1.7)
Years of education > 9	0.8 (0.3 – 2.3)	1.3 (0.7 – 2.4)	0.8 (0.3 – 1.8)	1.2 (0.8 – 2)
**Employment**				
Unemployed	1	1	1	1
Employed/retired	0.5 (0.1 – 1.6)	0.8 (0.4 – 1.5)	0.8 (0.3 – 2.2)	1.2 (0.7 – 1.9)
Employer/private business	0.4 (0.1 – 1.3)	0.8 (0.4 – 1.5)	1.03 (0.3 – 3.2)	0.9 (0.41 – 1.8)
**Density index (DI)**				
>2.3	1	1	1	1
1.5 – 2.3	0.4 (0.2 – 0.8)*	0.9 (0.6 – 1.4)	0.5 (0.3 – 0.9)*	0.7 (0.5 – 1.1)
<1.5	0.4 (0.2 – 0.8)*	1 (0.6 – 1.6)	0.6 (0.3 – 1.1)	0.9 (0.7 – 1.4)
**Marital status**				
Married	1	1	1	1
Not married	1.5 (0.6 – 3.9)	1.2 (0.7 – 2.1)	0.4 (0.2 – 0.7)*	0.6 (0.4 – 0.9)*
**Number of chronic health problem score**				
None	1	1	1	1
One	1.5 (0.7 – 3.5)	2.1 (1.5 – 3.1)*	3.4 (1.8 – 6.5)*	17 (1.2 – 2.5)*
Two or more	6.7 (3.1 – 14.4)*	3.1 (1.9 – 4.9)*	4.8 (2.6 – 9.2)*	1.9 (1.3 – 2.7)*
**Self-perceived functional disability and affection score**				
0–9 little disability	1	1	1	1
10–16 moderate	3.3 (1.3 – 8.5)*	2.9 (2 – 4.3)*	5.9 (1.9 – 17.9)*	3.4 (2.3 – 4.9)*
17–36 sever disability	11.9 (4.8 – 29.2)*	5.2 (3.4 – 7.9)*	22.2 (7.7 – 63.9)*	5.8 (3.9 – 8.5)*
**Body mass index (BMI) score**				
<25	1	1	1	1
(25 – 30)	0.7 (0.3 – 1.5)	0.9 (0.7 – 1.5)	0.7 (0.4 – 1.5)	0.7 (0.5 – 1.04)
>30	0.5 (0.2 – 1.2)	1.1 (0.7 – 1.7)	0.6 (0.3 – 1.3)	0.8 (0.5 – 1.1)
**Physical activity score**				
Low < 1	1	1	1	1
Middle (score 1–3)	0.4 (0.2 – 0.8)*	0.6 (0.4 – 0.8)*	0.9 (0.6 – 1.5)	0.9 (0.7 – 1.3)
High >3	0.1 (0.02 – 0.4)*	0.4 (0.2 – 0.7)*	0.4 (0.1 – 1.2)	0.7 (0.4 – 1.1)
**Smoking status **(non smoker as reference)	2.6 (1.2 – 5.6)*	1 (0.72 – 1.4)	0.8 (0.4 – 1.3)	1
**Have someone who supports when needed **("no" as reference)	0.5 (0.2 – 0.9)*	1.1 (0.7 – 1.7)	0.9 (0.5 – 1.8)	1.3 (0.8 – 1.9)
**Have someone shares happiness & sorrow **("no" as reference)	1.5 (0.5 – 4.2)	0.8 (0.5 – 1.4)	0.4 (0.2 – 0.8)*	0.8 (0.5 – 1.3)

* *p *<*0.05 *according to multinomial logistic regression analysis with all variables studied entered into the model. Note that because education, employment, and density index are part of the SES score, two models were build in one the total SES score was entered, and in the other the three individual components were entered.

## Discussion

We found that a higher proportion of women reported poor health compared to men, a finding in line with studies from other developing countries (Pakistan, Bangladesh) and countries in transition (Ukraine) [15,16,19,20]. Macintyre et al., reported from Britain that the pattern of gender differences in health outcomes (including SRH) is highly complex and varies across the life course [21]. This potentially explains why the female disadvantage has not been found in some countries [22,23]. The same authors reported that a female excess in psychological distress was consistently apparent across the life course. Low psychological well-being is associated with poorer SRH [24]. Evidence from Aleppo suggest that mental distress is common in low income women [25], and that anxiety and depression are is more prevalent among women than men [1]. This also may explain why women who have some social support were less likely to report poor SRH compared to men.

We found age to be a significant predictor of poor SHR in both men and women in Aleppo. Evidence from other countries shows that gender differences in SRH are age dependent [21,26].

Our findings suggest that being married is an important predictor of poor SRH in women. Married women were more likely to report poor health than unmarried women and more than twice as likely as married men to report poor SRH. Whilst our survey does not provide direct explanation for this discrepancy, it is reasonable to assume that this may have its roots in gender roles and traditions of the Syrian society. Married women bear the burden of household duties and child care whilst at the same time having less opportunity to do recreational activities. Such burden may be less pronounced among better off families, which may explain why socioeconomic status was a more important predictor of SRH in women compared to men. Women in the higher socio-economic group were more than three times less likely to report poor health compared to those in the lower socio-economic group. Still, this is an assumption that warranted further research.

Gender roles and societal traditions may also reflect on the observed association between obesity and physical activity with SRH. High BMI scores showed a significant association with poor SRH in both men and women, however, the effect did not reach statistical significance when adjusting for other covariates. Ferraro et al. reported that people with a BMI of more than 30.5 reported poorer rating of health than those with normal or below normal weight even after controlling for a number of indicators of ill health and physical functioning [27]. A recent study on obesity in Aleppo showed that obesity was higher in women than in men (46.3% vs. 28.4%, P < 0.001) with the highest prevalence being in the older age group (46–65) [4]. Currently obesity is not stigmatized in Syria as in western nations. On the contrary, many sections of Syrian society still see obesity as a sign of prosperity.

In Aleppo, half of the women but only one fifth of men reported low levels of physical activity [4,27]. This is likely to be due to physical activity being more feasible for men than women in a generally conservative society. This may also explain why lack of physical activity was more strongly associated with poor SRH in men than in women, since certain recreational activities may not be an option for many women in the Syrian society. The finding in men is in line with evidence showing that sports participants reported better SRH than non sports participants [28].

In our study, smoking was associated with poor SRH among men, but not among women. Findings from other studies suggested an independent association between smoking and SRH with never smokers rating their health best [14,29]. Smoking in Syria, like in many Arab countries, is traditionally a male activity [3]. Cigarette smoking among men has recently reached very high proportions, with 51% of adult men in Aleppo being daily smokers [3]. Men daily smokers in Syria consume on average twice as women [3], which can help explain the predominance of reporting poor SRH by male smokers.

This study is not without limitations. First, the cross sectional nature of the data limited our ability to understand causal mechanisms that result in poor SRH in men and women. For example, it was not clear whether low psychological wellbeing among women results in poorer SRH or whether poor health outcomes result in a higher level of depressive symptoms in females. Second, increased gender disparity of poor SRH in the older ages in our study may result from confounding introduced by what is termed 'mortality selection', especially at older ages [19]. Adult men have higher age-specific mortality rates than females in most societies. The highest degree of confounding usually occurs at older ages where mortality rates among males are higher than those of females thus leaving a group of men who are healthier than their female counterparts [19].

Despite these limitations, our study is the first in an Arab country to report on SRH and its determinants in men and women. We have interpreted the findings in light of previous research on smoking and obesity as well as our understanding of the unique attributes of the Syrian society and culturally shaped gender roles. There is evidence that self ratings of health and morbidity are influenced by differences in expectation, perception, social experience and comparison all of which may vary throughout time but are culturally shaped [26,30,31]. We suggest that further qualitative and quantitative studies are needed in Arab country in order to provide a fuller understanding of the mechanisms that result in poor SRH in men and women in Syria and alike societies.

### What this paper adds

• This study is the first in an Arab country to report on SRH and its determinants in men and women.

• The findings strongly support evidence from other developing countries which shows that females are much more likely than males to report poor SRH.

• Our findings suggest that certain determinants of SRH (i.e. marital status, physical activity, social support) may be culturally shaped in that they reflect specific gender roles and social norms and expectations.

### Policy implications

Women are particularly prone to reporting poor SRH, which can reflect subtle societal traits related to prevailing norms and gender roles. In-depth studies are needed to provide a fuller understanding of what appears to be culturally influenced determinants of SRH. This is important when designing and delivering culturally sensitive and effective interventions in Syria and similar Arab societies

## Conclusion

Poor SRH is more pronounced among women and in older ages on Syria. Some of the determinants of SRH in Syria, such as those related to age and chronic condition/disability, are likely to reflect life processes that do not differ between countries and societies. Other findings such as those on the relation to marital status and physical activity are likely to reflect specific gender roles and social norms and expectations. These will be of prime importance when designing interventions to combat health problems such as obesity, smoking, and mental distress in Syria. Poor married women in particular, seem to be burdened by their duties and left with little opportunity for recreation, which in turn results in poor SRH. This requires concentrated efforts to better understand and intervene to improve the physical and psychological wellbeing of women in the Syrian and similar Arab societies.

## Competing interests

The author(s) declare that they have no competing interests.

## Authors' contributions

TA designed the study, performed the statistical analysis, wrote the final draft, and revised the manuscript. BA participated in designing the study and writing the first draft. SR participated in performing the statistical analysis. TPM participated in revising the manuscript. KDW participated in revising the manuscript. WM participated in the planning of analysis and revising the manuscript.

## Pre-publication history

The pre-publication history for this paper can be accessed here:


